# Glutamine metabolism in diseases associated with mitochondrial dysfunction

**DOI:** 10.1016/j.mcn.2023.103887

**Published:** 2023-08-15

**Authors:** Rebecca Bornstein, Michael T. Mulholland, Margaret Sedensky, Phil Morgan, Simon C. Johnson

**Affiliations:** a Center for Integrative Brain Research, Seattle Children’s Research Institute, Seattle, USA; b Department of Anesthesiology and Pain Medicine, University of Washington, Seattle, USA; c Department of Laboratory Medicine and Pathology, University of Washington, Seattle, USA; d Department of Neurology, University of Washington, Seattle, USA; e Department of Applied Sciences, Translational Bioscience, Northumbria University, Newcastle, UK

**Keywords:** Mitochondrial disease, Glutamine toxicity, Neurodegenerative disease

## Abstract

Mitochondrial dysfunction can arise from genetic defects or environmental exposures and impact a wide range of biological processes. Among these are metabolic pathways involved in glutamine catabolism, anabolism, and glutamine-glutamate cycling. In recent years, altered glutamine metabolism has been found to play important roles in the pathologic consequences of mitochondrial dysfunction. Glutamine is a pleiotropic molecule, not only providing an alternate carbon source to glucose in certain conditions, but also playing unique roles in cellular communication in neurons and astrocytes. Glutamine consumption and catabolic flux can be significantly altered in settings of genetic mitochondrial defects or exposure to mitochondrial toxins, and alterations to glutamine metabolism appears to play a particularly significant role in neurodegenerative diseases. These include primary mitochondrial diseases like Leigh syndrome (subacute necrotizing encephalopathy) and MELAS (mitochondrial myopathy with encephalopathy, lactic acidosis, and stroke-like episodes), as well as complex age-related neurodegenerative disorders such as Alzheimer’s and Parkinson’s diseases. Pharmacologic interventions targeting glutamine metabolizing and catabolizing pathways appear to provide some benefits in cell and animal models of these diseases, indicating glutamine metabolism may be a clinically relevant target. In this review, we discuss glutamine metabolism, mitochondrial disease, the impact of mitochondrial dysfunction on glutamine metabolic processes, glutamine in neurodegeneration, and candidate targets for therapeutic intervention.

## 1. Mitochondrial metabolism and glutamine

**Fig. 1.**
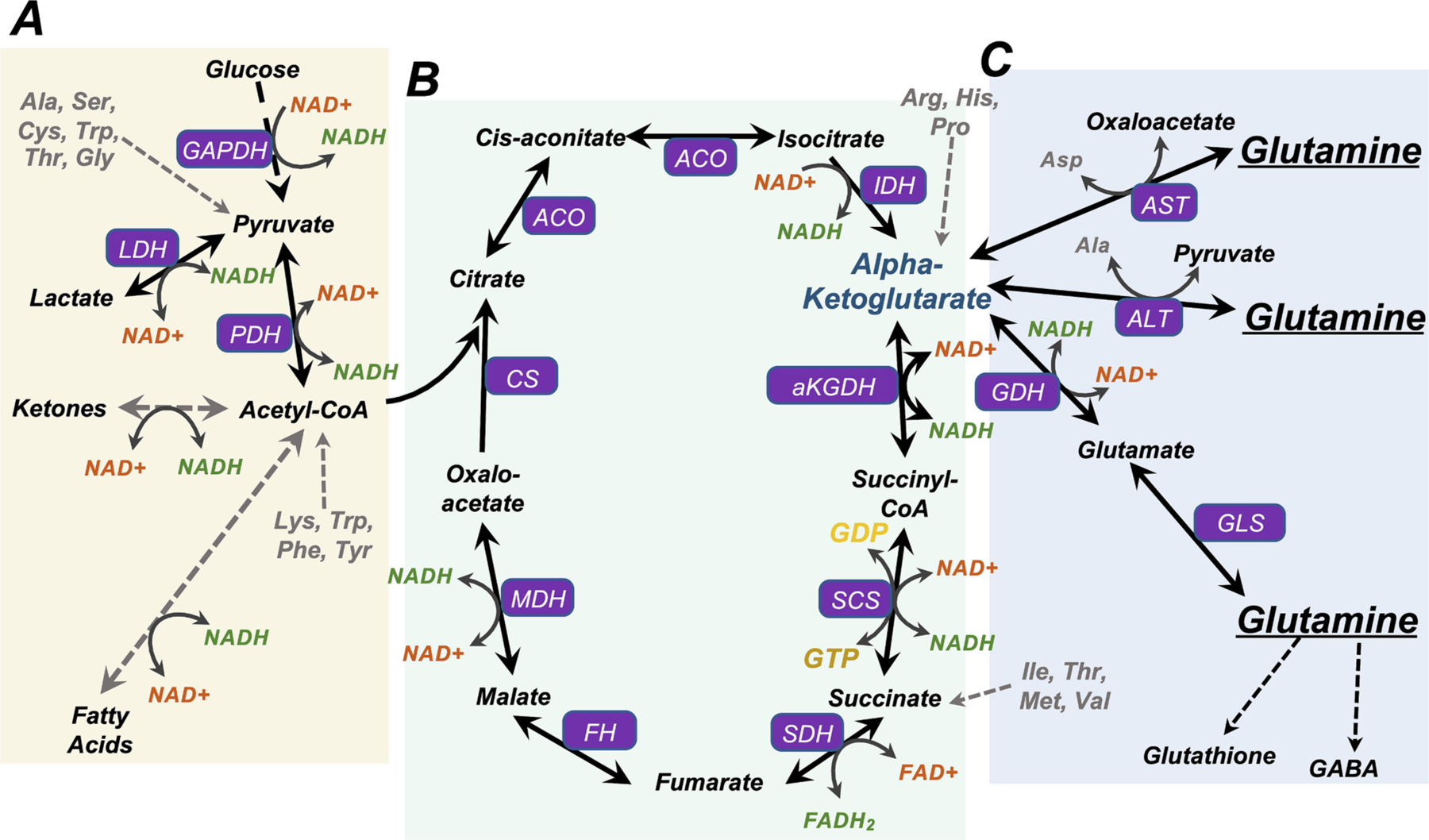
Glutamine and NADH in central metabolic pathways. (A) Catabolism of glucose, ketones, and fatty acids generates Acetyl-CoA. Glucose is broken down through glycolysis to form pyruvate. In settings of reduced flux through the TCA cycle, such as in anaerobic conditions or settings of increased NADH/NAD+, pyruvate generated through glycolysis can be reduced to lactate by LDH (lactate dehydrogenase) with concomitant oxidation of NADH to NAD+. Ketone catabolism converges at acetyl-CoA and NADH is produced from NAD+ in the process. Ketogenesis, the reverse process, primarily occurs in the liver, consuming acetyl-CoA and NADH. Beta-oxidation of fatty acids generates acetyl-CoA and NADH. Metabolism of the amino acids Lys, Trp, Phe, and Tyr also converges at acetyl-CoA, while Ala, Ser, Cys, Trp, Thr, and Gly can be metabolized to pyruvate. (B) An overview of the tri-carboxylic acid (TCA) cycle. Acetyl-CoA generated through glycolysis, ketone metabolism, fatty acid metabolism, lactate metabolism, and the metabolism of certain amino acids can feed the TCA cycle to sustain carbon flow and aerobic metabolism. NADH is generated from NAD+ at four steps in the cycle: isocitrate to alpha-ketoglutarate (aKG) catalyzed by isocitrate dehydrogenase (IDH), aKG to succinyl-CoA catalyzed by aKG dehydrogenase (aKGDH), succinyl-CoA to succinate catalyzed by succinyl-CoA synthetase (SCS), and malate to oxaloacetate catalyzed by malate dehydrogenase (MDH). (C) Central pathways of glutamine metabolism. Through the process of glutaminolysis, glutaminase (GLS) catalyzes the reaction of glutamine to glutamate, and glutamate is converted to aKG by glutamate dehydrogenase (GDH). Glutamine may also be converted directly to aKG by alanine or aspartate aminotransferase (ALT and AST), with concomitant production of alanine or aspartate, respectively. Glutamine is also a precursor to glutathione and GABA, which are critical for cellular antioxidant defenses and neurotransmission.

Mitochondria are the primary energy-generating organelles in most mammalian cells. Mitochondria orchestrate varied metabolic processes and provide adenine triphosphate (ATP), the primary chemical used for short-term cellular energy storage and transfer (McInnes, 2013). A greatly simplified summary of central mitochondrial metabolism is as follows (see Fig. 1): catabolic metabolism of carbohydrates, fatty acids (FAs), ketones, and amino acids (AAs) produces acetyl-CoA, which can enter the tricarboxylic acid (TCA) cycle. Metabolism of acetyl-CoA by the TCA cycle generates NADH and FADH_2_, which power pumping of protons across the mitochondrial inner membrane by the electron transport chain (ETC). This proton pumping generates a proton gradient across the inner membrane, which is harnessed by ATP synthase to drive ATP production (Kanter, 2018). Though some ATP is generated through glycolysis and substrate level phosphorylation in the TCA cycle, and more obscure processes, the ETC provides the bulk of ATP in most eukaryotic cells under normal conditions.

Glucose is a key molecule used for energy storage and transport in eukaryotes, and regulation of extracellular/circulating glucose is crucial in mammalian health. Extracellular glucose is transported into cells via GLUT/SLC2A family transporters. Most glucose imported into cells is either metabolized into pyruvate or incorporated into glycogen, a polysaccharide of glucose used for efficient osmo-normal storage. The major catabolic fates of pyruvate include conversion to acetyl-CoA; anaerobic metabolism to lactate by lactate dehydrogenase (LDH), producing ATP and oxidizing NADH to NAD+; or conversion to alanine by alanine aminotransferase (ALT) with a concomitant conversion of glutamine to alpha-ketoglutarate (Fig. 1A). Glycogen can be rapidly hydrolyzed to free glucose upon demand.

FAs provide an alternate form of energy storage. FAs are typically stored as triglycerides, three FA chains covalently attached to a glycerol molecule. Fat stored in lipid droplets can provide free FAs for metabolism, and lipolysis of triglycerides in adipose tissue provides free FAs and glycerol to the blood stream. These are transported bound to albumin and taken up by peripheral tissues for catabolism. Catabolism of fatty acids via beta-oxidation produces acetyl-CoA, available to enter the TCA cycle or be converted to ketones (Fig. 1A).

Acetyl-CoA can be used to generate ketone bodies through ketogenesis, providing an alternate circulating catabolic energy source. The majority of ketogenesis occurs in the liver, though neuroprotective ketogenesis has been reported to occur in astrocytes, a brain glial cell type important for neuronal metabolism, glutamine cycling (see below), and neuromodulation (Takahashi et al., 2014; Guzman and Blazquez, 2004; Guzman and Blazquez, 2001). Ketones are metabolized to acetyl-CoA by end organs and cells, such as neurons in the CNS. The brain is a major utilizer of ketones, which are a preferred energy source in the developing mammalian brain (Steiner, 2019; Yeh and Sheehan, 1985; Yeh et al., 1977). Evidence suggests ketone supplementation is beneficial in multiple neurologic diseases including epilepsy, traumatic brain injury, and Alzheimer’s disease (Jensen et al., 2020; Broom et al., 2019; Arisaka et al., 2020).

### 1.1. Glutamine metabolism

Amino acids (AAs) can also enter the TCA cycle, although most dietary AAs are first converted into glucose or ketones in the liver. Glutamine is the most abundant AA in both muscle and blood, and an important and versatile circulating and intracellular metabolite in mammals (Cruzat et al., 2018; Ling et al., 2019). In addition to involvement in protein synthesis, glutamine is a precursor for the antioxidant glutathione, which is critical for maintaining cellular redox status and protecting cells from oxidative damage, and glutamine acts as a key alternative substrate for supplying the TCA cycle and driving oxidative metabolism when glucose metabolism is disrupted (Newsholme et al., 2003; Chen et al., 2018). Glutamine also plays unique roles in neuronal activity, detailed below.

Glutamine is a key anaplerotic (TCA cycle-feeding) substrate (Brunengraber and Roe, 2006) (Fig. 1C). Glutamine is of particular importance to rapidly dividing cells, including neoplastic cells, feeding the TCA cycle, enabling protein synthesis, and providing a precursor for glutathione and for nucleotides necessary for cell replication (Liao et al., 2019; Bowtell and Bruce, 2002; Martins et al., 2020). Glutamine can enter the TCA cycle after conversion to glutamate and then alpha-ketoglutarate (aKG) through a process termed glutaminolysis, catalyzed by glutaminase (GLS) and glutamate dehydrogenase (GDH). Alternatively, alanine or aspartate aminotransferase (ALT and AST, respectively) can convert glutamine to aKG, with concomitant production of alanine or aspartate, respectively. A mitochondrial AST isozyme exists, which converts mitochondrial glutamine to aKG for TCA entrance. This AST reaction is a key part of the malate-aspartate shuttle, a key biochemical system for the net effect of moving transferring NADH equivalents from the cytosol to the mitochondria (increasing cytoplasmic NAD+ and mitochondrial NADH) (Borst, 2020). In addition, mitochondrial AST driven conversion of glutamate and oxaloacetate to aKG and aspartate has been found to be necessary for the survival of proliferating cells in the setting of ETC inhibition (Birsoy et al., 2015). ALT is expressed predominantly in the liver, while AST is expressed in multiple tissues, including brain (Kim et al., 2020).

## 2. Metabolic sequelae of mitochondrial dysfunction

### 2.1. NADH and metabolism

The impact of mitochondrial dysfunction on cellular respiration depends on the precise mitochondrial defect, and in many cases is poorly understood. Rewiring of central carbon metabolism is common among genetic defects impacting ETC function. ETC defects can slow consumption of TCA cycle generated NADH, resulting in an increase in the NADH/NAD+ ratio. Increased NADH/NAD+ inhibits flux through NADH redox sensitive steps in the TCA cycle and glycolysis. This situation results in decreased glucose metabolism with a compensatory increase in the products of anaerobic glucose use, including lactate and alanine (Mukaneza et al., 2019; Aspuria et al., 2014).

NADH redox (the ratio of NADH/NAD+) is a major regulator of cellular metabolism, with multiple core metabolic reactions influenced by this ratio (Fig. 1). In glycolysis, G3P dehydrogenase (GAPDH) catalyzes the production of 1,3-bisphosphoglycerate from glyceraldehyde-3-phosphate (G3P), with the reaction concomitantly generating NADH from NAD+. Pyruvate dehydrogenase (PDH) catalyzes conversion of pyruvate into acetyl-CoA while consuming NAD+ to generate NADH. In fat metabolism, oxidation of L-β-hydroxyacyl CoA’s, catalyzed by 3-hydroxyacyl-CoA dehydrogenase, is also an NAD+ consuming reaction. Within the TCA cycle itself, NADH is produced (and NAD+ consumed) in three reactions: 1) the conversion of isocitrate to oxalosuccinate by isocitrate dehydrogenase (IDH), 2) the conversion of aKG to succinate by aKG dehydrogenase (a-KGDH), and 3) the conversion of malate to oxaloacetate by malate dehydrogenase (MDH). IDH isoform IDH3 functions in the context of the TCA cycle and is NAD+ reactive, while IDH1 and 2 utilize NADP+.

Each of these NAD+ dependent reactions and are slowed or inhibited by an elevated NADH/NAD+ ratio. The impact is most pronounced at PDH and IDH: PDH catalyzes the rate-limiting step for entry of glucose carbons into the TCA cycle, and NAD+ dependent IDH catalyzes a rate-limiting and irreversible step of the TCA cycle (Gabriel et al., 1986). When NADH/NAD+ is high, conversion of pyruvate to lactate by lactate-dehydrogenase (LDH) is favored over conversion to acetyl-CoA, while TCA cycle flux through IDH is inhibited. LDH consumes NADH, replenishing NAD+, contributing to the favorability of this lactate production in the setting of high NADH.

**Fig. 2.**
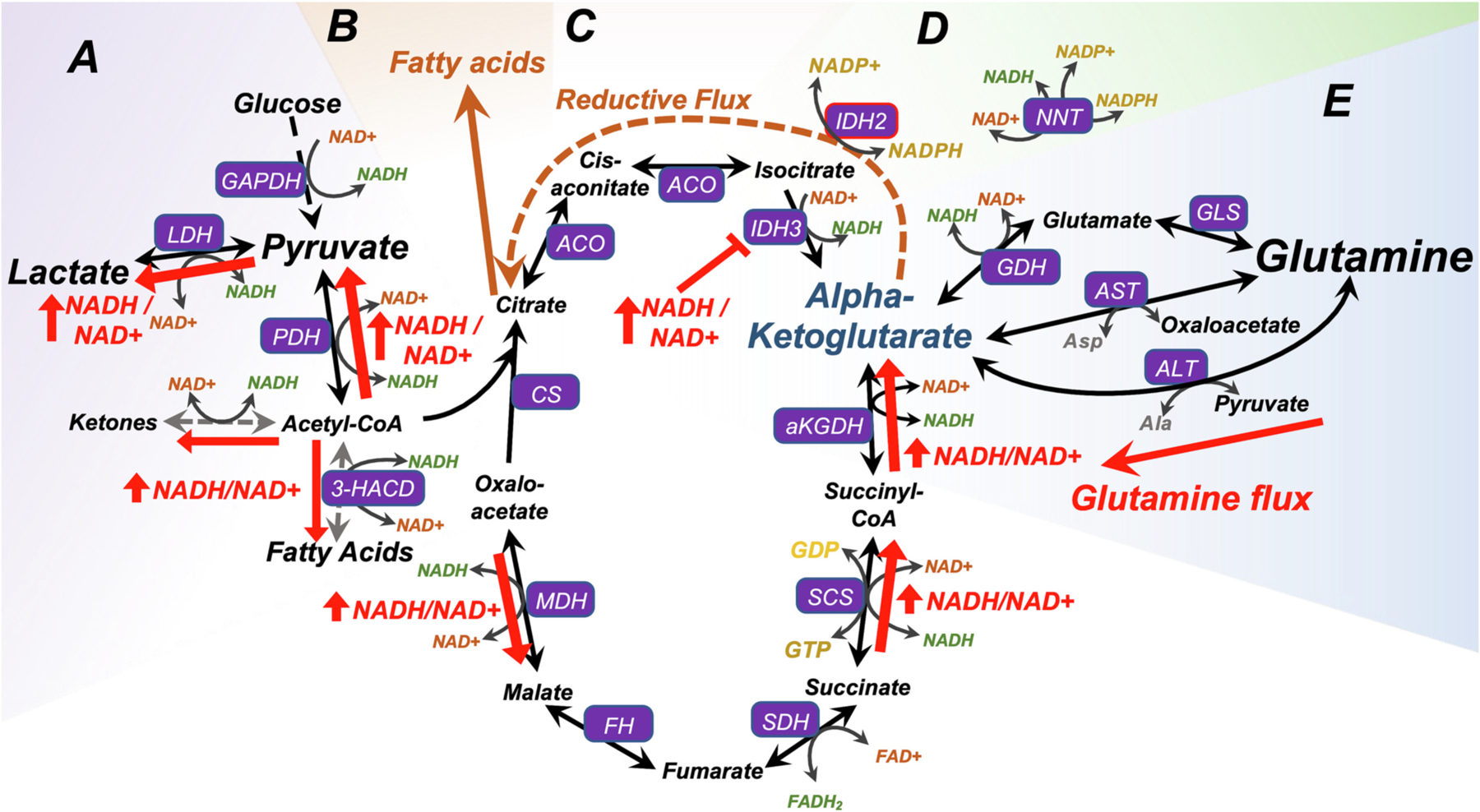
Tissue specific glutamine alterations in genetic mitochondrial disease. Reduced brain glutamine levels have been reported in the *Ndufs4*(KO) mouse model of Leigh syndrome, in MELAS patients, and in FA patients. Glutaminolysis enzymes have been found to be upregulated in the Purkinje cells of *Mfn2* deficient mice. Glutamine/glutamate/aKG have been shown to support mitochondrial oxidative phosphorylation in brain mitochondria from *Ndufs4* deficient mice where significant defects in ETC CI driven mitochondrial oxygen consumption are seen with other metabolic substrates, such as pyruvate and lactate. Glutamine levels are also reduced in OPA1 deficient patients. In contrast to these brain findings, glutamine levels are increased in both animal models of *COX10* deficiency, and in both human patients and animal models of MM, and IOSCA resulting from deficiencies in the mitochondrial helicase *Twinkle*. These changes are thought to result from increased muscle catabolism.

In conditions of increased NADH/NAD+, pyruvate can also be converted to alanine by alanine-aminotransaminase (ALT), concomitant with the consumption of glutamate and production of aKG (Figs. 1, 2). Increased NADH/NAD+ can impair carbon flux through the TCA cycle and leads to cataplerosis, the exit of intermediates from the cycle. Major cataplerotic products include pyruvate, aspartate, adenylosuccinate, and glutamate (Owen et al., 2002).

The production of glutamate from aKG is favorable as a cataplerotic outlet in some high NADH contexts as the reaction, catalyzed by GDH, can consume NADH and generates NAD+. GDH is also linked to TCA cycle function through regulation by guanosine triphosphate (GTP) levels – GTP, generated by succinyl-CoA synthetase, is a potent inhibitor of GDH (Mastorodemos et al., 2005). Mitochondrial dysfunction can impact glutamine levels differentially depending on relative changes to NADH redox and TCA cycle flux: de-inhibition by low GTP would be expected to promote either anaplerosis or cataplerosis, depending on NADH redox. The NAD+ concentration also plays a role in GDH regulation via the post-translational modification ADP-ribosylation, catalyzed by the mitochondrial ADP-ribosyl transferase (mART). NAD+ is utilized by mART to covalently and reversibly inhibit GDH, and low NAD+ results in reduced inhibition (Herrero-Yraola et al., 2001). Accordingly, when TCA disruption with high NADH/NAD+ impairs flux to aKG, these modes of regulating GDH drive increased glutamine flux into the TCA cycle via de-inhibition of this enzyme (Fig. 2).

Citrate is critical for fatty acid synthesis. Under conditions where citrate is depleted but NADH is not high, glutamine-derived aKG can both replenish citrate through IDH2 dependent reductive metabolism which consumes NADPH (Wise et al., 2011; Mullen et al., 2014) (Fig. 2). NADH produced by oxidative flux of aKG can replenish NADPH via interconversion by nicotinamide nucleotide transferase (NNT) (Mullen et al., 2014). This allows glutamine to provide carbons for driving both cellular metabolism and biosynthesis.

ETC CI, an NADH dehydrogenase and coenzyme Q reductase, is a major NADH consuming enzyme. Defects in ETC CI can lead to increased NADH/NAD+, resulting in disruption of NADH redox regulated processes detailed above. Through this mechanism, ETC CI dysfunction can drive increases in lactate and alanine, reduce glucose carbon flux to the TCA cycle, increase cataplerosis, and reduce entry of pyruvate into the TCA cycle. Interestingly, genetic defects in both ETC CI and PDH can cause the genetic mitochondrial disease Leigh syndrome (LS, detailed above) (De Meirleir, 2013); this seems to indicate a special role for pyruvate flux to acetyl-CoA in the pathogenesis of LS, though a clear explanation for the link is lacking.

### 2.2. Impacts of altered glutamine metabolism in settings of mitochondrial dysfunction

As detailed above, glutamine can enter the TCA cycle as aKG, an anaplerotic process. This can be increased in settings where altered NADH/NAD+ redox drives reductive catabolism. In this context, aKG produced through glutaminolysis can alleviate bottlenecks at IDH, replenish citrate, and relieve NADH accumulation (see Fig. 2).

This glutamine anaplerosis has been observed in multiple models of mitochondrial dysfunction. Cells with mitochondrial defects have an increased dependence on glutamine for proliferation and survival, and increased glutaminolysis is a critical compensatory mechanism in certain forms of mitochondrial dysfunction (Chen et al., 2018; Motori et al., 2020). Mitochondrial respiratory capacity appears to dictate whether flux at the aKG junction will be oxidative or reductive, where severe defects in respiratory capacity drive more reductive flux (Chen et al., 2018). In rapidly proliferating cells with mitochondrial defects, reductive TCA cycle flux is crucial in part due to its role in aspartate synthesis; aspartate is a precursor for both proteins (as an amino acid) and nucleotides, providing a backbone for de novo pyrimidines synthesis (Birsoy et al., 2015; Sullivan et al., 2015). For example, anaplerosis of glutamine is necessary for proliferation of PDH deficient cells (Roma et al., 2022; Wang et al., 2019).

The importance of carbon entry via aKG in mitochondrial dysfunction is demonstrated by mitochondrial oxygen consumption assays in the setting of ETC CI defects: in ETC CI deficient mitochondria, pyruvate/malate driven oxygen consumption is significantly impaired, but oxygen consumption is partly rescued when glutamate/malate are provided as substrates and fully rescued with aKG/malate (Kayser et al., 2016; Johnson et al., 2020). These findings show that aKG (and glutamine, as a precursor to aKG) can compensate for certain disruptions of the TCA cycle and/or PDH.

Consistent with these data in isolated mitochondria, dimethyl-KG (DMKG), a cell permeable KG, has been found to attenuate (albeit modestly) disease in the *Ndufs4*(KO) (Lee et al., 2019). The NAD+ precursor nicotinamide riboside (NMN) was also found to modestly modify disease course, and the authors proposed a model whereby attenuation of NADH/NAD+ redox increases aKG and this attenuates disease progression (though redox changes were only detected in skeletal muscle). In support of this model, recent data has shown that expression of the yeast NADH dehydrogenase Ndi1 in the neurons of *Ndufs4*(KO) mice, using Nestin-Cre driven expression, can rescue NADH/NAD+ redox and many symptoms of disease (McElroy et al., 2020). Whether alterations to glutamine/glutamate/aKG metabolism play an important causal role in the link between altered redox and disease remains to be more directly demonstrated, but all evidence would appear to support such a connection.

Entry of glutamine into the TCA cycle as aKG has also been shown to enhance mTORC1 signaling, which is known to occur in the setting of mitochondrial disease (Yang et al., 2017; Johnson et al., 2019). The causal link between glutamine, aKG, mTOR, is complex; the role of mTOR in mitochondrial disease in whole organisms appears to be defined more by immune activity than metabolism, but cultured cell models support a cell-autonomous role for mTOR dysregulation, and this remains an active area of research (Johnson et al., 2019; Johnson et al., 2013; Stokes et al., 2022; Capristo et al., 2022).

## 3. Mitochondrial dysfunction and human disease

Mitochondrial dysfunction is an umbrella term used to refer to a variety of distinct defects in normal mitochondrial functions. Parameters of mitochondrial dysfunction include altered mitochondrial enzyme level or enzymatic activity, including in the electron transport chain (ETC) complexes; mitochondrial network alterations, including hyper-fragmentation or hyper-connectivity resulting from defective mitochondrial fission or fusion; mitochondrial structural or substructural abnormalities, such as abnormal cristae morphology or swelling; altered mitochondrial biogenesis or turnover, which can be related to changes in mitophagy; abnormal reactive oxygen species (ROS) production or neutralization; impaired function of the TCA cycle or related metabolic processes; and impaired ability to generate ATP, either overall capacity or from a specific substrate (Montgomery and Turner, 2015; Bornstein et al., 2020).

### 3.1. Genetic mitochondrial disease

Defects in mitochondrial DNA (mtDNA) or nuclear genes encoding mitochondrial factors can lead to genetic mitochondrial diseases. Many distinct forms of mitochondrial disease, grouped and defined based on clinical presentation, have been described. These include Leigh syndrome (LS), the most common pediatric presentation of genetic mitochondrial disease; Mitochondrial Encephalomyopathy Lactic Acidosis and Stroke-like Episodes (MELAS); Friedrich’s ataxia; Kearns-Sayre syndrome (KSS); and Leber’s Hereditary Optic Neuropathy (LHON) (Breuer et al., 2013; Orsucci et al., 2021; Frazier et al., 2019). While individually rare, mitochondrial diseases are the leading cause of inborn errors of metabolism, as well as a leading causes of genetic neurological dysfunction. Mitochondrial diseases are typically clinically defined with named syndromes representing constellations of symptoms which can arise from lesions in one of multiple genetically distinct loci. In LS, for example, more than 85 distinct genes have been causally associated with the disease, with many distinct disease causing variants in some of these genes (Kim et al., 2022).

Clinical presentation among genetic mitochondrial diseases is strikingly heterogenous; for example, LHON is primarily a single organ disorder, while LS is a complex multi-system disease impacting many organ systems. In general, there is no clear functional mechanism distinguishing genes causing one form of mitochondrial disease versus another. No current models exist to explain which genetic lesions will lead to which clinical manifestations.

### 3.2. Mitochondria in complex human diseases

Genetic lesions with weak functional consequences can contribute to complex multigenic diseases. Genetic loci associated with mitochondrial components have been linked to many such diseases through genome-wide association studies (GWAS) (Bornstein et al., 2020; Johnson et al., 2017; Gonzalez et al., 2023; Johnson et al., 2015). Mitochondrial dysfunction is thought to play a causal role in a wide range of human pathologies, from cancer to normative aging. Neurodegenerative diseases, in particular, have been widely associated with altered mitochondrial functions. Links between mitochondrial function and complex neurologic diseases are reviewed in detail elsewhere, but a discussion focused on the role of altered glutamine metabolism in some key diseases is provided in below.

### 3.3. Environmental toxins

Mitochondrial dysfunction can also result from environmental exposures to chemotherapeutics, pollutants, mitotoxic compounds found in foods or dietary supplements, and agricultural chemicals such as pesticides. Environmental toxins can impact mitochondrial phospholipids, damage mtDNA, inhibit mitochondrial enzymes including the ETC, or cause mitochondrial membrane depolarization (the latter best typified by the early 20th century diet drug 2,4-DNP) (Meyer et al., 2013; Gorini et al., 2018; Zolkipli-Cunningham and Falk, 2017; Grundlingh et al., 2011).

A small group of particularly well documented environmental mitotoxins have been directly linked to human neurodegenerative diseases. These include the ETC complex I (ETC CI) inhibiting pesticide/piscicide rotenone and mitochondrial ROS generating herbicides paraquat and diquat, both linked to Parkinson’s disease through both epidemiologic and pre-clinical studies (Spivey, 2011; Olubodun-Obadun et al., 2022; Miyazaki et al., 2020; Tanner et al., 2011; Tangamornsuksan et al., 2019; Drechsel and Patel, 2009; Yan et al., 2016). The ETC CI inhibiting plant toxin annonacin has similarly been linked to progressive supranuclear palsy (PSP), a disease with similarities to both PD and LS (Spivey, 2011; Olubodun-Obadun et al., 2022; Miyazaki et al., 2020). Ample experimental evidence links these and other mitochondrial toxins to various neurodegenerative disease forms in model systems.

Overall, these mitotoxins provide some of the most compelling evidence for mitochondrial origins of neurodegenerative disease: animal studies causally demonstrate mitochondrial toxin exposure leads to disease, while human epidemiologic data provides strong links between environmental exposure and disease risk. It is worth noting that although links between mitochondrial toxins and neurodegenerative disease are robustly supported by both animal and human studies, multiple well known mitotoxins continue to receive renewed approval for agricultural use. A good example is antimycin A, a compound used in the laboratory as a potent ETC CIII inhibitor and employed as a pesticide (specifically as a piscicide in fish management and aquaculture, see EPA 738-R-07-007).

## 4. Altered glutamine levels in mitochondrial disease

While roles for glutamine in metabolic responses to mitochondrial dysfunction in cultured cells are clear, the precise impact of mitochondrial defects is likely highly cell, tissue, and context specific. As with many cell-based findings, it is not entirely clear where or when reductive flux occurs in vivo, and what functional tipping points are important for NADH redox, aspartate synthesis, etc.

Some cell-specific data in LS and Friedrich’s Ataxia (FA, caused by defects in mitochondrial iron handling) implicate glutamine/glutamate metabolism in the pathogenesis of these diseases arising from mitochondrial dysfunction. In the *Ndufs4* model of LS, cell-specific depletion of the ETC CI subunit *Ndufs4* in glutamatergic neuron (using the VGlut2-Cre promoter) reproduces nearly the entire phenotype of whole-body *Ndufs4*(KO) model, including CNS lesions (Johnson et al., 2020; Bolea et al., 2019), while total neuron loss via Nestin-Cre mediated excision yields similar results (Quintana et al., 2010). In FA, VGLUT-positive terminals in the dentate nucleus are selectively depleted, while the majority of neurons are spared (Koeppen et al., 2011). In these two forms of mitochondrial dysfunction, disease appears to uniquely impact glutamatergic neurons.

Metabolomic studies have shown glutamine levels are depleted in specific tissues in certain mitochondrial diseases. *Ndufs4*(KO) brains are significantly deficient in glutamine, glutamate, and aKG in all brain regions (Johnson et al., 2013; Terburgh et al., 2021). Similarly, patients with MELAS and FA have reduced brain glutamate, and patients with hereditary optic atrophy type 1 caused by OPA1 mutations have reduced plasma glutamate (Moller et al., 2005; Huxtable et al., 1979; Chao de la Barca et al., 2019). In each of these diseases, the neuronal tissues are the most significant target of mitochondrial dysfunction.

**Fig. 3.**
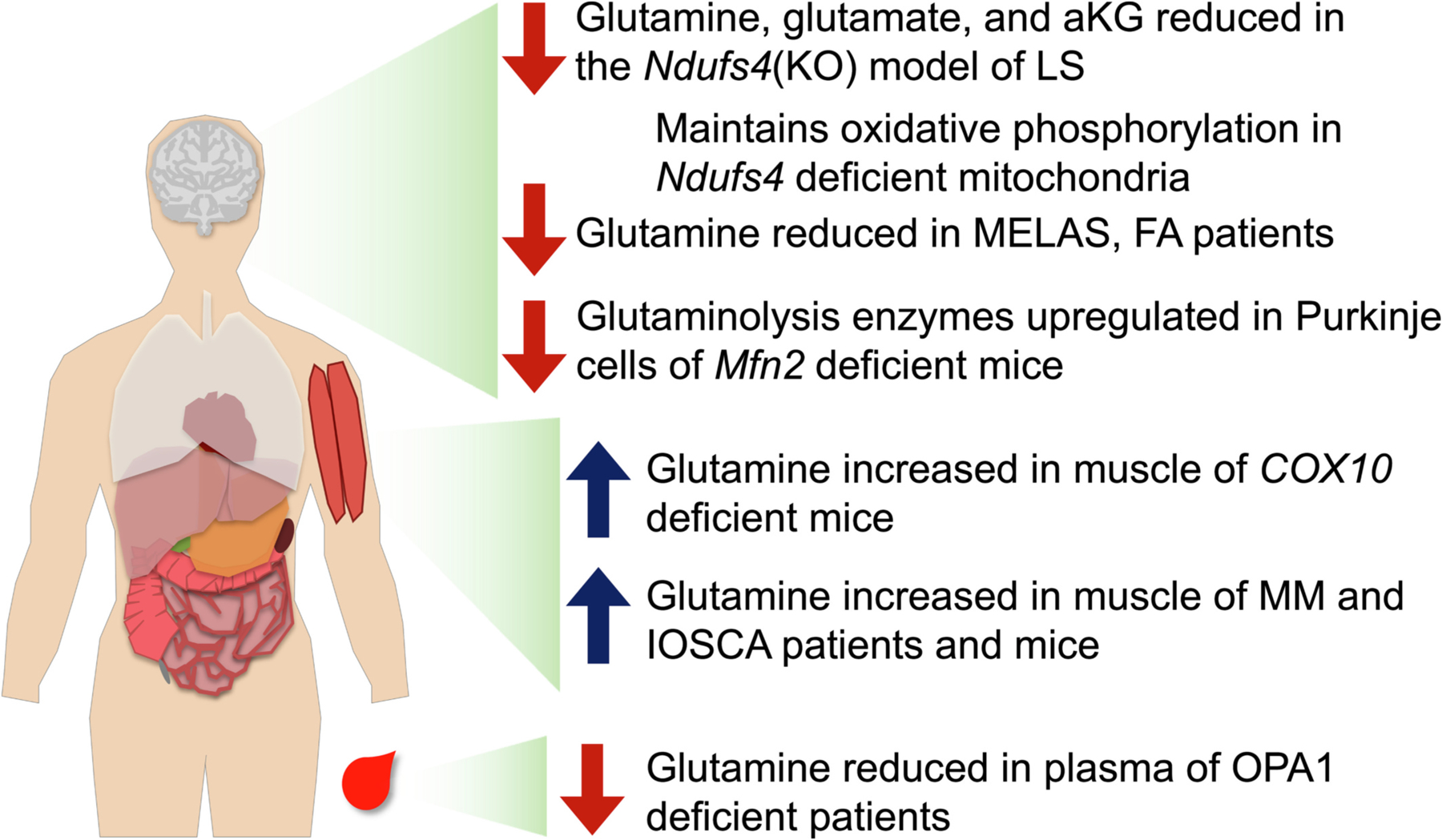
Glutamine metabolism in the setting of mitochondrial dysfunction. (A) Increased NADH/NAD+ resulting from ETC CI dysfunction can drive increased pyruvate and lactate levels by driving LDH and PDH reactions in an NAD+ regenerating direction. (B) Fatty acid synthesis is critical for cellular proliferation and, in some cases, function and survival. (C) Anaplerotic flux of glutamine into the TCA cycle both through oxidative and reductive flow can sustain fatty acid synthesis. (E) Mitochondrial NAD+ dependent IDH (IDH3) is the TCA cycle step most sensitive to inhibition by high NADH/NAD+, and the IDH3 reaction is generally irreversible. In conditions of high NADH, aKG can be metabolized to citrate via reductive flow that takes advantage of NADP+ dependent IDH. NNT can catalyze the interconversion of NADPH and NADH to regenerate NAD+. (E) Glutamine can provide anaplerotic input into the TCA cycle at aKG via multiple mechanisms, including conversion through ALT or AST, concomitant with the production of alanine or aspartate, or through conversion to glutamate then to aKG by way of glutaminase and glutamate dehydrogenase. Aspartate is a key nucleotide precursor, and production from glutamine is key to survival proliferating cells with mitochondrial defects. By both providing both NADH relief through reductive flux and sustaining flow through the TCA cycle downstream of IDH3 the glutamine/glutamine/aKG pathway can sustain mitochondrial oxidative metabolism in the face of altered NADH/NAD+ redox resulting from ETC dysfunction.

In contrast to these findings, defects causing mainly muscle pathology generally appear to *increase* free glutamate in muscle and/or plasma. In the muscle specific *COX10* (heme A:farnesyltransferase cytochrome *c* oxidase assembly factor) knockout mouse model of mitochondrial myopathy (MM), the amino acids (AAs) glutamine, glutamate, and alanine are significantly elevated in muscle, but not plasma, brain, or liver (Chen et al., 2018). Changes were attributed to muscle wasting, though it was unclear why glutamine, glutamate, and alanine were specifically elevated among AAs. In MM and infantile onset spinocerebellar ataxia (IOSCA) resulting from distinct mutations in TWINKLE (the replicative mitochondrial DNA helicase), patients show increased plasma glutamate (Nikkanen et al., 2016). Mouse models for these TWINKLE defects were found to have distinct skeletal muscles AA changes - all AAs were increased in the MM model, while only lysine, arginine, glutamine, and glutamate were elevated in the IOSCA model (Fig. 3).

The relationship between changes in brain and muscle glutamine, glutamate, and related metabolites have not yet been directly explored, and even indirect evidence is lacking as MM models have tended to focus on muscle, and neurogenerative disease models on brain.

### 4.1. Metabolic dysfunction in mitochondrial disease

Systemic metabolic abnormalities are a common feature of genetic mitochondrial disease, with direct or indirect relations to glutamine. Glucose homeostasis, in particular, is impacted in multiple forms of mitochondrial dysfunction. Mitochondrial diabetes (mtDB) is a prominent clinic feature in some mitochondrial diseases including maternally inherited diabetes and deafness (MIDD), Kearns-Sayre syndrome (KSS), MELAS, and FA; an estimated ~22 % of all mitochondrial disease patients presenting with mtDB (Ashfaq et al., 2021). Unlike type 1 diabetes, caused by immune-mediated loss of pancreatic β-cells, or type 2, caused by insulin resistance in glucose consuming tissues, dysregulation of glucose homeostasis in mtDB appears to occur when mitochondrial dysfunction leads to an impairment of β-cell insulin secretion (Schaefer et al., 2013).

In addition to mtDB, in which glucose clearance from the bloodstream is impaired, hypoglycemia with normal hepatic function also often occurs in mitochondrial disease. The etiology of mitochondrial disease associated hypoglycemia is not well understood (Ashfaq et al., 2021; Mochel et al., 2005). In addition to pancreatic defects, central glucose sensing may be altered in both mitochondrial diabetes and mitochondrial hypoglycemia. Cell autonomous and systemic glucose regulation are impacted by mitochondrial dysfunction, but the precise relationship between cell-autonomous and systemic metabolic features of mitochondrial disease are often unclear.

Products of anaerobic respiration of glucose - pyruvate, lactate, and alanine - are frequently elevated in the blood of patients with mitochondrial disease (Smeitink et al., 2006; Smeitink, 2003). Increased lactate can also be detected in impacted brain regions in some mitochondrial diseases, such as Leigh syndrome, via proton magnetic resonance spectroscopy (MRS), contributing to clinical diagnosis (Lin et al., 2003; Chi et al., 2011; Delonlay et al., 2013). Hyperlactemia and lactic acidosis are severe acute metabolic sequelae in mitochondrial disease patients, but it is not clear whether increased levels of tissue or circulating lactate, pyruvate, or alanine play any causal role in the pathogenesis of progressive (non-acute) symptoms (Maassen et al., 2004). These metabolites are elevated in the *Ndufs4*(KO) mouse model of ETC CI Leigh syndrome, and levels are attenuated by disease-modulating treatments, suggesting some role in disease (Johnson et al., 2020; Johnson et al., 2013; Stokes et al., 2022; Bornstein et al., 2022).

The cellular origins of systemic metabolic derangements in mitochondrial disease are not easily established. For example, while muscle is the largest producer of lactate in normal conditions, recent data suggests that immune cells are the major source of blood lactate in at least certain settings (Stokes et al., 2022; Stokes et al., 2022). In other situations, such as altered glutamate and aKG in the brain of *Ndufs4*(KO) mice, the cellular source of metabolic dysfunction has not been established, and the diversity of, and complex relationships between, brain cell types complicates experimental approaches (Johnson et al., 2020).

## 5. Glutamine in the brain

### 5.1. Brain metabolism

The mammalian brain has unique metabolic requirements and energetic demands. In humans, the brain consumes more energy than any other organ in the body by mass, and is estimated to account for ~25 % of all glucose use and ~20 % of oxygen consumption (Steiner, 2019; Goyal and Raichle, 2018). Energetically costly neuronal activities include generation of transmembrane potentials through ion pumping, trafficking of neurotransmitter vesicles at the synapse, and synthesis of neurotransmitters (Mergenthaler et al., 2013; Tomasi et al., 2013; Du et al., 2008).

**Fig. 4.**
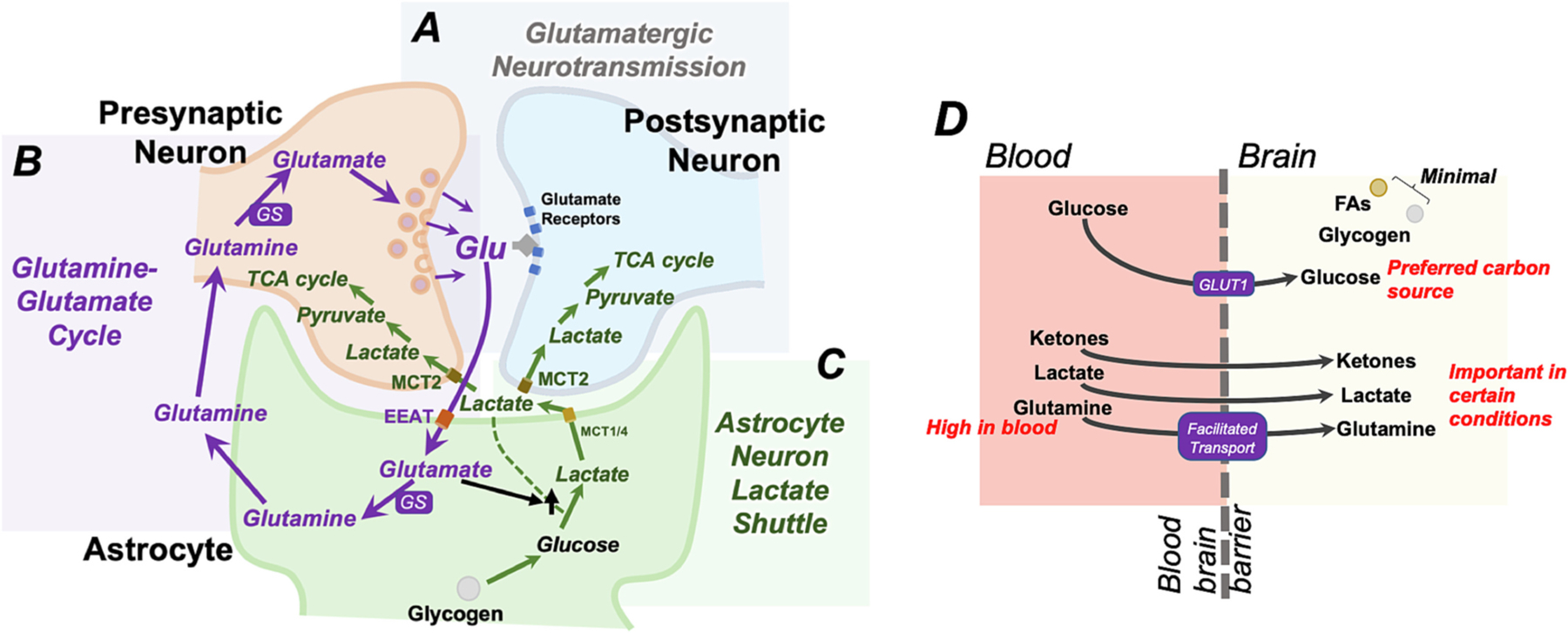
Glutamine in the brain. (A) Glutamate is the most abundant excitatory neurotransmitter. Glutamate in pre-synaptic glutamatergic neurons is packaged into vesicles at the synapse. Vesicular glutamate released into the synaptic cleft is acts on glutamate receptors at the postsynaptic neuron. (B) The astrocyte-neuron glutamine-glutamate cycle ensures that glutamate in the synapse is rapidly removed from the synapse to allow for subsequent synaptic events. In this cycle, glutamate imported into astrocytes through the EEAT glutamate transporters. Within astrocytes, glutamate is converted to glutamine, which is not a neurotransmitter, by glutaminase (GS). This is subsequently trafficked back to presynaptic neurons, where it is converted back into glutamate and re-packaged into synaptic vesicles to repeat the cycle. (C) Lactate produced in astrocytes can be exported via astrocyte-specific monocarboxylate transporters (MCT1 and MCT4) to subsequently be imported into neurons through MCT2 and supply the TCA cycle and oxidative metabolism. This process is termed the astrocyte neuron lactate shuttle (ANLS). Some evidence indicates that astrocyte lactate production is stimulated by intracellular glutamate levels and by glutamate receptors at the membrane, possibly linking ANLS flux to glutamatergic synaptic activity. (D) The brain has minimal local energy stores in forms of glycogen, which can be hydrolyzed to generate glucose, and lipid droplets, which can generate free fatty acids (FAs). The majority of brain metabolism is supported by circulating glucose, largely transported through the blood brain barrier by the glucose transporter GLUT-1. Ketones and lactate are important carbon sources for brain metabolism under certain conditions such as fasting/starvation and in the setting of strenuous exercise, respectively. Ketones are also an important metabolite during brain development. Glutamine is the most abundant amino acid in the blood and can feed central carbon metabolism. Glutamine concentrations in the brain are tightly regulated; glutamine is imported into the brain via facilitated and sodium dependent transport mechanisms.

The brain has limited supplies of glycogen and lipid droplets (lipid-rich organelles) compared with other tissues (Fig. 4). FAs make up approximately half of the brain’s mass, but most are structural components rather than energy stores (Bruce et al., 2017; Barber and Raben, 2019). Some lipid droplets are present in astrocytes, microglia, and neurons (Farmer et al., 2020). Lipid droplets in astrocytes can support neurons by providing stores for oxidation to ketones for uptake by neurons (Barber and Raben, 2019). Lipid droplet accumulation in microglia is primarily associated with inflammation, rather than energetic stores (Farmer et al., 2020; Marschallinger et al., 2020).

Glycogen is present in astrocytes, and glucose released from glycogen can be converted to lactate to provide fuel to the TCA cycle in neurons via the astrocyte-neuron lactate shuttle (ANLS). Astrocyte lactate is transported out of astrocytes via monocarboxylate transporters 1 and 4 (MCT1 and MCT4) and into neurons via MCT2. Astrocyte lactate generated in this manner appears important in hypoglycemia or periods of intense neuronal activity (Falkowska et al., 2015; Sinadinos et al., 2014; Brown and Ransom, 2007). While various details of the ANLS are hotly debated (reviewed elsewhere, see (Magistretti and Allaman, 2018)), some evidence indicates astrocyte lactate production may be stimulated by astrocyte glutamate receptors and intracellular astrocyte glutamate concentrations, linking the ANLS to glutamatergic neuron activity. Supply of carbons to the TCA cycle in glutamatergic neurons via the ANLS is thought to preserve pentose-phosphate pathway mediated glucose metabolism, which generates NADPH and the nucleotide precursor ribose-5-phosphate, and provide cataplerotic flow into the TCA cycle without consumption of amino acids.

Given the minimal fuels stores, the brain requires constant supply via circulation (Fig. 4). The blood brain barrier (BBB) is impermeable to many metabolites, but allows glucose entry, mainly through the glucose transporter GLUT-1, and AA’s through a variety of transport systems (Keaney and Campbell, 2015; Zaragoza, 2020). Glucose is the primary carbon source for the mammalian brain under most conditions, but ketone bodies, lactate, and glutamine are also major substrates for metabolism. Metabolite preference is strongly influenced by nutritional status, age, health, and other physiologic factors. During strenuous physical activity, for example, lactate generated by skeletal muscle crosses the BBB and is catabolized in the brain. Ketone bodies, on the other hand, are present at low levels in the blood in adults, but high in neonates and in the settings of dietary ketosis and starvation (Proia et al., 2016; Riske et al., 2017). Circulating ketones are high during the neonatal growth period and a preferred substrate for the neonatal brain. In the developing brain, ketones serve as direct precursors for lipid synthesis during this period of active myelination, as well as a primary circulating energy source (Yeh and Sheehan, 1985; Cotter et al., 2011; Edmond et al., 1985; Stokes et al., 2021).

Glutamine is the most abundant amino acid in the blood, at 10–100 times the concentration of other amino acids (Cruzat et al., 2018; Ling et al., 2019). Glutamine can cross the BBB via facilitated transport and sodium-dependent transport, allowing dietary glutamine, and glutamine produced by muscle proteolysis, to supply the brain. Uptake is controlled at the abluminal membrane (brain-ward facing side of the BBB cells) to regulate CNS levels (Smith et al., 1987; Hawkins, 2009; Lee et al., 1998).

### 5.2. Glutamate neurotransmission and the glutamine/glutamate cycle

In addition to providing an energy source, glutamine is a precursor for glutathione, a key molecule involved in protection from oxidative damage, and is a precursor for both glutamate and GABA, the two most abundant neurotransmitters in the brain. Glutamate is a critical neurotransmitter: it is reported that more than 90 % of neurons have glutamate receptors and almost 40 % of neurons are classified as glutamatergic (Gasiorowska et al., 2021). While glutamine concentrations are about ten times higher than glutamate in the blood, glutamate is most abundant AA in the brain. Glutamate is highly concentrated in CNS tissue at 10,000–12,000 μmol/L, compared with 50–100 μmol/L in blood. However, for brain homeostasis and function, glutamate in extracellular spaces must be maintained at only 0.5–2 μM.

Synaptic glutamate transiently increases to millimolar concentrations during synaptic neurotransmission, and rapid removal of glutamate is critical to limit the length of signal at the synapse and allow for subsequent synaptic events (Conway, 2020; Wang and Zhang, 2005; Wang and Reddy, 2017). Astrocytes remove glutamate from the synaptic cleft and extracellular space and recycle it to neurons for re-use through a process called the glutamine-glutamate cycle (Fig. 4). Excitatory amino acid transporters (EAATs) transport synaptic glutamate into astrocytes, with EAAT2 responsible for ~90 % of transport (Kim et al., 2011). Within astrocytes, glutamate is then converted into the nonneurotransmitter glutamine by glutamine synthetase (GS), providing an intermediate for transport back to neurons. Glutamine export from astrocytes is facilitated by the glutamine transporter SN1 (Broer et al., 2002). Extracellular glutamine is imported into neurons via glutamine transporters such as SAT1 and SAT2 (Yao et al., 2000). Neuronal glutamine is subsequently used to synthesize glutamate via the enzyme glutaminase, whereafter it can be packaged into presynaptic vesicles for reuse (Conway, 2020).

The glutamine/glutamate cycle provides control over the length of stimulation and proper function is critical not only for neural activities, but also to prevent glutamate excitotoxicity, which is thought to result from chronic or over-stimulation of glutamate receptors (discussed below). Tight regulation of glutamine metabolism plays an important role in maintaining optimal intracellular and extracellular glutamate concentrations. Glutamate conversion to aKG is an anaplerotic reaction, as detailed, but can also contribute to regulation of overall glutamate levels.

## 6. Glutamine metabolism and neurodegenerative disease

### 6.1. Neurodegenerative mitochondrial diseases

Impaired glucose metabolism in the brain is a well-documented phenomena in genetic mitochondrial diseases. Patients with LS, MELAS, Friedrich’s ataxia (FA), and MM show reduced cerebral glucose metabolism and a shift from oxidative respiration to aerobic glycolysis by position emission tomography (PET) and carbon 13-labeled MRS (Lindroos et al., 2009; Haginoya et al., 2016; Otsuki et al., 2005; Molnar et al., 2000). Similarly, there is a significant increase (interpreted as an accumulation) of glycolytic intermediates in the brain of the *Ndufs4* (KO) mouse model of ETC CI deficiency, neurodegeneration, and LS (Johnson et al., 2013).

Dysregulation of brain glutamine metabolism has also been reported in the setting of mitochondrial dysfunction. Glutamine, glutamate, and aKG are significantly reduced in all brain regions in the *Ndufs4*(KO) mouse model of CI deficient LS, and dimethyl-KG modestly attenuates disease in this model (Johnson et al., 2020; Lee et al., 2019; Johnson et al., 2013). Glutaminolysis is increased in neurons with oxidative phosphorylation deficiency, and glutaminolysis enzymes are upregulated in Purkinje cells isolated from the *Mfn2* deficient mouse model of mitochondrial dysfunction show upregulation (Motori et al., 2020).

These data suggest that glutamine/glutamate/aKG support the TCA cycle in the brain when flux from glucose and through IDH is perturbed, a model directly supported by evidence mitochondrial oxygen consumption data. Pyruvate and glutamate driven complex I dependent (rotenone inhibited) mitochondrial oxygen consumption is 30–40 % reduced in isolated mitochondria from *Ndufs4*(KO) brain, but aKG fully supports normal maximal complex I dependent (rotenone inhibited) respiratory capacity (malate was included in each case) (Kayser et al., 2016; Johnson et al., 2013). Glutamate itself only partly rescued oxygen consumption in these experiments, likely due to the lack of cytoplasm (where glutamine synthetase is located) in the mitochondrial preparations. A comprehensive explanation for these findings remains to be determined, but they reveal that flux (at least in the ex vivo setting) through aKG can continue in a setting where ETC CI dysfunction has led to impairment of flux through PDH and IDH (Porpaczy et al., 1987).

### 6.2. Complex neurodegenerative diseases

Extensive research has been dedicated to studying the role of metabolism in complex neurodegenerative diseases with strong links to mitochondrial dysfunction. These include Alzheimer’s (AD), Parkinson’s (PD), amyotrophic lateral sclerosis (ALS), multiple sclerosis (MS), and others (Bornstein et al., 2020; Johnson et al., 2017; Han et al., 2021; Barcelos et al., 2019). Patient and animal studies reporting decreases in cerebral glucose utilization by MRI and PET, reduced expression of glucose transporters, decreased levels of glycolytic proteins, and strong associations between alterations in glucose metabolism and associated neuropathology and dementia. Glutamine mishandling is also a well-documented in complex neurodegenerative diseases. The following is a brief overview of evidence linking glutamate metabolism to these diseases.

#### 6.2.1. Alzheimer’ disease (AD)

AD is a progressive age-related neurodegenerative disorder involving severe cognitive impairment and memory loss coincident with amyloid plaque accumulation in hippocampal and neocortical tissue (Magalingam et al., 2018). AD is not generally thought to be a direct result of metabolic derangement, but aberrant glutamine metabolism has been reported in AD, and altered glutamate neurotransmission appears to play some role in pathology (Wang and Reddy, 2017; Liu et al., 2019).

Cortical tissue taken from AD patients showed decreased glutamate with increased glutamine (Griffin and Bradshaw, 2017). MRS studies of AD patients reported decreased glutamate in living cortex (Fayed et al., 2011; Rupsingh et al., 2011). In mice, the CRND8 mouse model of AD, which predisposes mice for amyloid accumulation, showed both glutamate and glutamine deficiencies in multiple brain regions (Salek et al., 2010).

Glutamate excitotoxicity is thought to play a role in AD pathobiology (Conway, 2020; Wang and Reddy, 2017; Liu et al., 2019; Lakhan et al., 2013). Signaling via the glutamate sensing NMDAR (*N*-methyl-d-aspartate receptor, a glutamate responsive receptor) is necessary for neuronal function, but the receptor also plays a key role in Ca2+ influx mediated excitotoxicity when overstimulated, and both insufficient and over-active NMDAR signaling can have catastrophic consequences to neurons (Wang and Reddy, 2017; Liu et al., 2019). NMDAR hypo- and hyper-functioning have both been described in AD, with amyloid deposits thought to directly impact NMDAR activity, and NMDAR activity contributing to cell death (Wang and Reddy, 2017; Lakhan et al., 2013). Amyloid accumulation appears to lead to a decrease in expression of EAAT2 on astrocytes, impairing glutamate reuptake via the glutamate-glutamine cycle and contributing to NMDAR-mediated excitotoxicity (Huang et al., 2018; Scott et al., 2011). Available data suggests that in early-stage AD, NMDAR hyper-functioning causes glutamate excitotoxicity, while loss of NMDA receptors and associated neurons subsequently contributes to overall hypo-functioning in late-stage disease (Lakhan et al., 2013).

Memantine is an FDA-approved mild affinity antagonist for the NMDAR specifically developed to interfere with glutamate excitotoxicity, one of a few pharmacological therapies for AD (Matsunaga et al., 2018). Unfortunately, though early trials suggest memantine provides some benefit, results are mixed, and even when benefits are reported they are extremely modest (Matsunaga et al., 2018; van Marum, 2009; Li et al., 2019). Whether targeting glutamine metabolism or signaling represents a viable strategy toward meaningful intervention in AD remains to be shown.

#### 6.2.2. Parkinson’s disease (PD)

PD is one of the most common neurodegenerative disorders, involving loss of neurons in the substantia nigra, a brain region critical for production of dopamine (Magalingam et al., 2018; Zhang et al., 2019). In a subset of PD patients, impacted neurons accumulate intracellular inclusions called Lewy bodies (Spivey, 2011). Pharmacological treatment of PD symptoms involves dopamine receptor agonists and levodopa, a dopamine precursor, to compensate for dopaminergic signaling deficiencies (DeMaagd and Philip, 2015) (Spivey, 2011). While PD is most directly associated with dopaminergic neurons, glutamate may also contribute to neurodegeneration in PD. Evidence suggests that glutamate receptor expression and activity are dysregulated in PD, while administration of NMDAR antagonists in animal models reduces PD symptoms, such as akinesia and rigidy, and increases the effectiveness of levodopa (Zhang et al., 2019; Vanle et al., 2018; Ahmed et al., 2011; Greenamyre and O’Brien, 1991). However, clinical trials of an NMDAR antagonist in PD patients show limited efficacy, and glutamate likely only modifies disease (Zhang et al., 2019).

#### 6.2.3. Amyotrophic lateral sclerosis (ALS)

ALS is an adult-onset neurodegenerative disease characterized by gliosis and neuron degeneration in the motor cortex, brainstem, and spinal cord (Kumar et al., 2013). Studies have found increased glutamate in ALS patient blood and cerebral spinal fluid. Post-mortem ALS patient brain samples were found to have increased glutamate but unchanged glutamine, but MRS studies of patients reported increases in both glutamate and glutamine (Kumar et al., 2013; Perry et al., 1987; Plaitakis et al., 1988; Fiszman et al., 2010; Spreux-Varoquaux et al., 2002). Altered glutamate receptor expression and activity have also been described in ALS patients and in models of disease, and genome-wide association studies have reported associations between glutamatergic neuron specific genes and ALS risk (Rosenblum and Trotti, 2017; van Rheenen et al., 2021). Despite this circumstantial evidence, pharmacologic and genetic studies manipulating glutamate receptor activity and glutamate signaling have led to only modest attenuation of outcomes in mouse models of ALS (Wang and Zhang, 2005; Blasco et al., 2014; Milanese et al., 2014; Bonifacino et al., 2017; Brownell et al., 2015; Guo et al., 2003; Joo et al., 2007; Hogg et al., 2018). Accordingly, as with AD and PD, altered glutamine metabolism may play a secondary or modifying role in disease, but available evidence does not a support a primary causal role in ALS disease pathogenesis.

#### 6.2.4. Multiple sclerosis (MS)

MS is a neuroinflammatory disorder impacting the brain and spinal cord. While MS is most strongly associated with an autoimmune etiology that is not well understood, significant evidence supports a role for mitochondrial dysfunction in the underlying causes of this disease. Notably, MS occurs coincidentally with the primary genetic mitochondrial disease LHON (described above) significantly more frequently than would be expected by chance; the combined disease has been termed Harding’s syndrome or LHON-MS (Hanaford and Johnson, 2022; Cawley et al., 2010; Parry-Jones et al., 2008). Efforts to elucidate and target mitochondrial pathways involved in inflammation are an active focus of research in MS as well as primary mitochondrial disease and have been proposed to causally link them (Hanaford and Johnson, 2022; Bargiela and Chinnery, 2019).

As in AD and ALS, circumstantial evidence supports some role for glutamine metabolism in MS, though the evidence is conflicting. Multiple studies have reported that glutamine and glutamate are increased in MS brains by MRS and that this increase is the best predictor of disease, while another study reported elevated plasma glutamine in MS patients (Tisell et al., 2013; Polacek et al., 2019; Yang et al., 2021a). However, others have reported decreased glutamine and glutamate in MS brains by MRS (MacMillan et al., 2016; Sastre-Garriga et al., 2005). Multivariate supervised analysis of brain MRS and a review of MRS literature both suggest glutamine and glutamate are among the most altered metabolites in MS brain, but change directionality depends on disease severity, as well as the region of white matter analyzed (i.e. unaffected brain vs lesion). Accuracy of determination is strongly dependent on the quality of the data (magnetic field strength, analysis methods, etc.) (Swanberg et al., 2019; Swanberg et al., 2022).

Glutamate excitotoxicity has been shown to contribute to disease progression, and glutamine antagonism attenuates disease, in mouse experimental autoimmune encephalitis models of MS, but the relevance of these models to MS is not clear (Hollinger et al., 2019; Pitt et al., 2000). Unfortunately, memantine (described above) trials in MS patients to date indicate that targeting glutamate excitotoxicity does not lessen cognitive decline or alter disease course in MS (Turalde et al., 2020).

## 7. Conclusions

Glutamine is central player in a wide range of biological processes. Glutamine is the most abundant amino acid in both muscle and blood, and a significant contributor to circulating metabolic homeostasis. Glutamine is a precursor for synthesis of the critical antioxidant glutathione and the most common neurotransmitter, glutamate, as well as an intermediate in glutamate recycling in the neuron-astrocyte glutamine-glutamate shuttle. Glutamine is also a major anaplerotic molecule, feeding the TCA cycle via input at aKG, enabling TCA cycle flux and feeding core TCA cycle driven biosynthetic pathways in settings of altered redox or low oxygen. Given these varied roles, it is perhaps of no surprise that experimental evidence has implicated glutamine metabolism in a wide range of pathologies, in particular those already linked to mitochondrial dysfunction.

Altered glutamine levels, or the levels or activity of glutamine metabolism related enzymes, are associated with a wide range of human pathologies. However, little evidence supports direct causality in links between altered glutamine metabolism and pathology. Moreover, while basic research links glutamine to a range of diseases, glutamine-targeting interventions have generally lacked efficacy in vivo. Memantine, designed to target NMDAR mediated glutamate excitotoxicity, is the best example of a glutamine/glutamate targeting therapy brought to the clinic and has proven to have little impact on disease course in AD (Matsunaga et al., 2018; van Marum, 2009; Li et al., 2019). This seems to suggest that if glutamine plays a significant role in AD, excitotoxicity is not a major mediating process. Efforts to promote TCA cycle entry through the glutamine/glutamate/aKG route using DMKG have shown some promise in both cell and mouse models of mitochondrial disease, but clinical translatability has not been tested. The modest benefits in a well-controlled mouse study may indicate that clinical efficacy will prove difficult to demonstrate, as with memantine.

The exception to the above statements is the setting of cancer, not discussed in this manuscript but the subject of multiple high-quality reviews (Souba, 1993; Li and Le, 2018; Li et al., 2021; Yang et al., 2021b). In cancer, reliance on glutamine catabolism for nucleotide precursors in rapidly dividing cells is a metabolic derangement amenable to pharmacologic targeting. Unfortunately, it is much easier to target a specific dysregulated metabolic process for inhibition with the aim of killing target cells than it is to promote intracellular utilization or to inhibit normal usage without significant off-target effects. The tissue and cell specificity of glutamine/glutamate metabolism in the brain, and extreme temporal and spatial specificity of glutamine/glutamate in glutamatergic synapse sub-structures, should give pause to any attempts to alter glutamine/glutamate metabolism through untargeted systemic approaches.

In summary, glutamine is pleiotropic molecule of key importance to a wide range of biological processes including antioxidant defense, neurotransmission, and cellular metabolism, and alterations in glutamine levels or metabolism have been reported in a wide range of diseases. However, causality is not often clear, and therapeutically beneficial targeting of glutamine metabolism has proven difficult. In the setting of neurodegenerative diseases, future efforts aimed at targeting therapies to neurons, or even synapses, may find efficacy where untargeted therapies have failed. In the setting of genetic mitochondrial disease, targeting glutamine-related metabolic pathways has shown some modest pre-clinical efficacy, but clinical transl. Ongoing work aimed at identifying additional therapeutic targets in glutamine metabolism and improving current intervention approaches may yield clinically relevant advances. Further study is needed to better understand the role and therapeutic potential of glutamine in human disease, including the cell and tissue specific roles of this metabolite.

## Data Availability

No data was used for the research described in the article.
